# Inoculation With Ectomycorrhizal Fungi and Dark Septate Endophytes Contributes to the Resistance of *Pinus* spp. to Pine Wilt Disease

**DOI:** 10.3389/fmicb.2021.687304

**Published:** 2021-08-06

**Authors:** Honglong Chu, Haihua Wang, Yanan Zhang, Zhumei Li, Chunyan Wang, Dongqin Dai, Ming Tang

**Affiliations:** ^1^State Key Laboratory of Conservation and Utilization of Subtropical Agro-bioresources, Guangdong Key Laboratory for Innovative Development and Utilization of Forest Plant Germplasm, College of Forestry and Landscape Architecture, South China Agricultural University, Guangzhou, China; ^2^College of Biological Resource and Food Engineering, Center for Yunnan Plateau Biological Resources Protection and Utilization, Qujing Normal University, Qujing, China; ^3^College of Forestry, Northwest A&F University, Yangling, China; ^4^Department of Food Science and Technology, College of Agriculture and Biotechnology, Chungnam National University, Daejeon, South Korea

**Keywords:** pine wilt disease, ectomycorrhizal fungi, dark septate endophyte, rhizosphere, microbial community, disease resistance

## Abstract

Pine wilt disease (PWD) is a deadly disease to pines (*Pinus* spp.) worldwide. The occurrence of PWD can reduce the relative abundance of root ectomycorrhizal fungi (ECMF) and dark septate endophytes (DSE). However, the effects of exogenous ECMF/DSE inoculation on the rhizosphere microbial community structure of *Pinus tabulaeformis* infected by pine wood nematode (PWN) is little known. Here, we tested how ECMF/DSE may improve resistance to PWD by quantifying microbial carbon biomass and soil enzymatic activity among different treatments at 6 and 9 months after PWN infection. Denaturing gradient gel electrophoresis (DGGE) was used to study the microbial community structure at 3, 6, and 9 months after PWN infection in the rhizosphere of *P. tabulaeformis* seedlings inoculated with ECMF/DSE. The results showed that exogenous ECMF/DSE inoculation reduced the disease severity caused by PWN infection. After PWN infection, the rhizosphere microbial carbon of seedlings inoculated with *Amanita vaginata*, *Suillus bovinus*, *Gaeumannomyces cylindrosporus*, and *Paraphoma chrysanthemicola* was 38.16, 49.67, 42.11, and 96.05% higher than that of the control group, respectively. Inoculation of ECMF/DSE inhibited the decrease of rhizosphere microbial biomass caused by PWN infection. The richness and diversity of *P. tabulaeformis* rhizosphere fungi at 9 months were reduced by PWN infection but partially recovered by the exogenous fungi (ECMF/DSE) inoculation except for *P. chrysanthemicola*, which indicates a role of ECMF/DSE in maintaining stability of the microbial community. Inoculation with ECMF/DSE increased the beneficial bacterial (*Thauera* sp., *Mesorhizobium* sp., etc.) and fungal groups (*Tomentella ellisii*, *Wilcoxina mikolae*, etc.) of in the rhizosphere. In summary, exogenous ECMF/DSE inoculation could increase *P. tabulaeformis* resistance to PWD probably by improving the rhizosphere microenvironment.

## Introduction

Pine wilt disease (PWD) is a severe threat to pine forests globally and typically kills affected trees within a few months (Mota and Vieira, 2008; Futai, 2013). The outbreak leads to irreversible changes to forest ecosystems and results in huge economic losses due to large reduction of timber, increased costs of management and disease control, and restriction of international trade (Mota and Vieira, 2008; Vicente et al., 2012). Pinewood nematode (PWN), *Bursaphelenchus xylophilus* (Steiner & Buhrer) Nickle, the causal agent of PWD was first described in 1934 in Louisiana, is originally from North America (Steiner and Buhrer, 1934; Nickle et al., 1981). Under natural conditions, PWD is transferred from infected trees to healthy trees by pine sawyer beetles (*Monochamus* spp.) while they feed on bark and foliage or during oviposition on susceptible pines. In addition, pinewood nematodes can be spread when humans transport infected wood, branches, and products to new areas. Human-mediated spread is not restricted by natural barriers and can occur very rapidly. Despite many years of research elucidating the mechanism of pathogenicity and modeling the spread of PWD (Mota and Vieira, 2008; Takemoto and Futai, 2008; Zhao et al., 2008; Futai, 2013; Yazaki et al., 2017), it is still difficult to treat PWD effectively; just like human “cancer,” no effective treatment is currently available, making PWD a constant threat to *Pinus* sp. plantations worldwide (Futai, 2013; Yazaki et al., 2017; Silva et al., 2021).

The rhizosphere is the plant roots-soil interface where interactions between microorganisms can influence plant growth, biogeochemical cycling, and tolerance to biotic and abiotic stress (Philippot et al., 2013). Rhizosphere microbiotas can even play a role in regulating the composition and biomass of plant communities in natural ecosystems (Kardol et al., 2007; Schnitzer et al., 2011; Meena et al., 2017). Just as the intestinal microflora affects human health, the root microbial community structure affects the health, growth, and development of plants (Berendsen et al., 2012; Cho and Blaser, 2012). Beneficial root microbial communities can promote plant growth and development and enhance plant resistance to disease and stress, and, similarly, less favorable microbial communities can inhibit plant growth and development and reduce plant disease resistance (Mendes et al., 2011). Exploitation and regulation of microorganisms in rhizosphere are widely recognized as a potential approach to integrated pest management for plant disease control (Mendes et al., 2011; Shi et al., 2015; Bach et al., 2016; Ab Rahman et al., 2018; Thomashow et al., 2019). Marfetán et al. (2020) found that seven fungal and four bacterial isolates from the *Austrocedrus chilensis* rhizosphere could inhibit mycelial growth of the pathogenic fungus *Phytophthora austrocedri*, and two of the bacterial isolates were able to reduce the symptoms in *A. chilensis* seedlings inoculated with *P. austrocedri*. Riaz et al. (2021) isolated 41 bacteria from the rhizosphere of different vegetables and, using pea (*Pisum sativum* L.) as a common background for assessing the effects of the bacteria, they found that rhizobacteria (*Bacillus* spp.) enhanced pea seedling growth and reduced the severity of root rot caused by *Fusarium solani*. Soil enzymes come from plant roots, soil microorganisms, and plant and animal residues and can be an indicator of soil potential to support biochemical processes, such as the decomposition of organic residues and nutrient cycling in the soil (Zhou et al., 2012; Raiesi and Salek-Gilani, 2018). Phosphatase plays an important role in the availability of soil phosphorus, while urease can hydrolyze urea and is closely related to soil organic matter content and the number of microorganisms. Invertase activity is related to the number of soil microorganisms and soil respiration (Guan et al., 1986). Soil enzyme activities and microbial biomass are closely related to soil health and can be affected by disturbances, such as plant disease (Alkorta et al., 2003; Dotaniya et al., 2019). And “good quality soil” can improve plant viability and disease resistance too (Mendes et al., 2011; Berendsen et al., 2012; Carrión et al., 2018; Kumar and Verma, 2019). So, the dynamic of community/structure of rhizospheric microbe and rhizospheric soil enzymes is supposed to be involved in plant health.

Mycorrhiza, including ectomycorrhizal fungi (ECMF) not only improve nutrient conditions, disease resistance, and stress tolerance of their plant hosts (Smith and Read, 2010), but also recruit and enrich other microorganisms that are beneficial to themselves or/and plants (Cameron et al., 2013; Gupta and Aggarwal, 2018). For example, mycorrhization helper bacteria (MHB), which play an important role in mycorrhiza, can increase the survival or germination rate of fungal spores; stimulate the growth of hyphae before the symbiotic stage, and enhance the reception of fungal signals by roots, stimulate the recognition of hyphae and roots; regulate physicochemical properties, etc. (Deveau and Labbé, 2017; Romero-Perdomo et al., 2021). At the same time, these microorganisms can also promote plant growth and improve disease resistance (Pieterse et al., 2014). Zhang et al. (2017) found that early contact between *Pinus tabulaeformis* and ECMF could improve plant growth and resistance to damping-off. Gonthier et al. (2019) found that mycorrhizal *Pinus sylvestris* was significantly less susceptible to *Heterobasidion annosum* than non-mycorrhizal plants; however, ectomycorrhizas differ in how effectively they protect hosts challenged by congeneric fungal pathogens. Like mycorrhizal, dark septate endophytes (DSE) have a wide distribution and similar functions to the host plant (Jumpponen and Trappe, 1998; Mandyam and Jumpponen, 2005; He et al., 2019). They colonize the epidermis and cortex of plant roots inter- and intra-cellularly and form characteristic structures of melanized hyphae and microsclerotia (Mandyam and Jumpponen, 2005). Poveda et al. (2020) found that *Brassica oleracea* var. *acephala* (kale) root endophytes were able to activate a systemic resistance in the plant against the bacterial pathogen *Xanthomonas campestris* and confer resistance against *Mamestra brassicae* larvae.

Our previous work showed that PWD and nematode infection altered the structure and abundance of root-associated fungi and reduced the density of mycelium in the rhizosphere, the biomass of fungi in the root tips, and the colonization rate of ECMF and DSE (Chu et al., 2016, 2018). Exogenous ECMF/DSE inoculations could improve the survival of pine trees suffering from PWN infection likely by mitigating the dysfunction of water (Chu et al., 2019). But how ECMF/DSE inoculations affect the rhizosphere microflora of pine trees suffering from PWN infection is unknown. To understand how ECMF/DSE may improve pine tree resistance to PWD, we investigated how the exogenous inoculation of ECMF/DSE affects the structure of rhizosphere microbial communities of *P. tabulaeformis* seedlings suffering from PWN infection. We used denaturing gradient gel electrophoresis (DGGE) as well as characterization of soil rhizospheric soil enzymes and microbial biomass carbon.

## Materials and Methods

### Plant, Fungal, and Nematode Materials

The fungi applied in the experiment were obtained from Northwest A&F University College of Forestry Microbiology Laboratory. The dark septate endophytes (DSE) *Gaeumannomyces cylindrosporus* (Gc) and *Paraphoma chrysanthemicola* (Pc) were isolated from the roots of *Astragalus adsurgens* Pall, which grew naturally on Qiandongshan lead-zinc mine tailings, Fengxian county, Shaanxi province, China (106°38'E, 33°49'N; Ban et al., 2012). ECMF species of *Suillus bovinus* (Sb) and *Amanita vaginata* (Av) were isolated from sporocarps harvested from the Huoditang forest region in Qinling Mountains in China (108°21'-108°29'E, 33°18'-33°18'N; Tang et al., 2008). These ECMF and DSE were selected because of their availability and record of intensive study (Tang et al., 2008; Ban et al., 2012, 2017; Zhang and Tang, 2012; Zhang et al., 2017; Chu et al., 2019). The inoculum of the ECMF and DSE strains was made according to Chu et al. (2019). In a brief, ECMF and DSE strains were cultured in half-strength potato dextrose agar solid medium for 2 weeks. Five agar disks of mycelium (5 mm) from each strain then were inoculated in a 250 ml Erlenmeyer flask containing 80 ml potato dextrose broth (PDB), with 15 replicates per strain. These flasks were incubated on a shaker at 120 rpm at 25°C for 20 days in the dark. Twenty days after fungal incubation, the mycelium was filtered out of the PDB using a fine, sterile mesh (230 mesh, 63 μm). The mycelium was then rinsed with sterile distilled water five times, and equal weights of fresh hyphae (18 g) were applied in the next step. Fungal inocula were prepared in a laminar flow hood by transferring the mycelium to a blender cup (Joyoung JYL-C022, Joyoung Company Limited, Jinan, China) and disrupting for 40 s in 500 ml of sterile, distilled water (Chu et al., 2019). The concentration of each inoculum was between 10^5^ ~ 10^6^ CFU/ml by dilution spread into PDA.

Pine seedlings (3 years old) were purchased from the Northwest A&F University Seed Co., Shaanxi Province, China. On November 16th, 2012, seedlings of similar size were selected and individually cultivated in pots (34 cm × 29 cm × 23 cm) that contained 4 L of growth substrate. The pinewood nematode (*B. xylophilus*) suspension inoculum was made according to Chu et al. (2019). Pine wood nematodes were cultured (PWN) on PDA plates covered with a colony of *Botrytis cinerea* at 25°C for 5 days, isolated by Baermann funnels, and then collected by centrifugation at 3000 rpm for 5 min. The nematodes were washed three times with sterile distilled water before inoculation. The density of nematode suspension was counted using a compound microscope (Chu et al., 2019).

### Experimental Design and Growth Condition

The growth substrate was soil collected from 5 to 30 cm depth from a nursery forest planted with *P. tabulaeformis* run by the Nursery of Forestry College, Northwest A&F University in Yangling, Shaanxi Province, China. The soil type is cinnamon and contained 18.58 gkg^−1^ organic matter, 35.78 mgkg^−1^ available nitrogen, 11.32 mgkg^−1^ available phosphorus, 158.56 mgkg^−1^ available potassium, and a pH (measured at a soil: water ratio of 1.0:2.5) of 7.4. The soil was air-dried, ground, and passed through 2 mm sieve to remove large stones and plant debris, then completely homogenized.

The experiment was conducted in the greenhouse of Northwest A&F University in Shaanxi province, China (34° 15' 59" N, 108° 03' 39'' E). The air temperature was between 24 and 35°C, the day light length varied from 12 to 14 h, and the relative air humidity was 55–78%. Each pot was irrigated once a week with 200 ml Hoagland nutrition solution (Hoagland and Arnon, 1950) throughout the growth period. The experiment consisted of a two-factor factorial design: exogenous ECMF/DSE inoculation (exogenous ECMF/DSE-inoculated plants and non-inoculated plants) and PWN infection (PWN-inoculated and non-inoculated plants). Each treatment had 30 pots of pine seedlings, and a total of 300 pots were planted {[4 kinds of exogenous fungi (ECMF and DSE) inoculation treatments + 1 control treatment] × 2 (PWN-inoculated and non-inoculated plants) × 30 = 300}.

Three months after the pine seedlings were transplanted to pots, uniformly sized seedlings were selected and the treatment pots were inoculated with two ECMF and two DSE, while the control pots of the ECMF/DSE treatment were inoculated with an autoclaved mixture mycelium suspension from all strains. Exogenous fungi inoculation was done by drilling four holes of 10 cm depth and 1.25 cm diameter with a hole punch 5 cm from the east, west, south, and north of the plant stem. Each hole was filled with 25 ml of the suspended inoculum, for a total of 100 ml mycelium suspension per pot. Three months after ECMF/DSE inoculation, PWN was inoculated at the lower end of the second lateral branch of the pine seedling. An inverted T-shaped wound was cut with a scalpel, and a small amount of absorbent medical cotton was inserted into the cut wound to prevent drying. 200 μl (about 8,000 heads) of PWN suspension was injected in the wound, and the wound was immediately wrapped with parafilm. 200 μl of sterile deionized water was injected into intra-group controls. At 2 weeks, 1 and 3 months after PWN inoculation, the disease incidence and mortality of pine seedlings were counted according to seedlings symptom with pine needles wilting and yellowing (Mota and Vieira, 2008). The rhizosphere soil from three randomly selected alive seedlings per treatment was collected by an auger (30 cm × 50 mm) at 3, 6, and 9 months after PWN inoculation, for a total of 90 samples. The rhizosphere soil was divided into two parts, one part was stored at 4°C for the determination of soil enzyme activity and microbial biomass carbon (C mic), and the other part stored at −20°C for the extraction of total DNA.

### Microbial Biomass Carbon Determination

Soil microbial biomass carbon was measured within 1 week after sampling according to Zhang et al. (2010). C mic was determined by chloroform-fumigation extraction (Vance et al., 1987). 25 g of soil was fumigated with alcohol-free CHCl_3_ for 24 h at 25°C in the dark. Both fumigated and non-fumigated samples were extracted with 0.5 molL^−1^ K_2_SO_4_ and filtered by Xinhua filter paper no.101 (Shuang Quan, China). Subsequently, the soluble organic carbon in fumigated and non-fumigated samples was determined. The results were calculated with the following formula (Zhang et al., 2010):

$$Cmic=\frac{EC}{K_{EC}}$$

where EC is the difference between carbon extracted from fumigated and un-fumigated soils, and *k*_EC_ (0.45) is the extraction efficiency coefficient. Cmic (μgkg^−1^ dry soil) is expressed on a dry soil basis (105°C, 24 h) and is the mean of three replicates (*n* = 3).

### Soil Enzymatic Activities Measurement

Dehydrogenase activity was assayed as described by Casida et al. (1964) and expressed in terms of the amount of triphenyl formazan (TPF) produced h^−1^ g^−1^ of soil (mg TPF g^−1^h^−1^) with reference to a standard curve of TPF. Urease activity was determined by the buffered method (Guan et al., 1986). Enzymatic activity was expressed as released NH_4_-N g^−1^ soil per hour (mg NH_4_-N g^−1^h^−1^) at 30°C measured colorimetrically using indophenol blue with reference to a standard curve of NH_4_-N. Invertase activity was determined colorimetrically using 3, 5-dinitrosalicylic acid and expressed as glucose mg g^−1^soil per hour at 37°C (Glc g^−1^h^−1^) with reference to a standard curve of glucose. Phosphatase activity was determined colorimetrically using disodium phenyl phosphate and was expressed as mg released phenol g^−1^ dry soil per hour at 37°C (mg PhOH g^−1^h^−1^) with reference to a standard curve of phenol (Guan et al., 1986).

### Denaturing Gradient Gel Electrophoresis Analysis

#### DNA Extraction and Nested PCR

DNA was extracted from the soil samples and stored at −20°C using the E.Z.N.A. Soil DNA Kit (Omega, United States) following the protocol of the manufacturer. The purity and concentration of DNA were measured by a SmartSpec^™^ Plus spectrophotometer (Bio-Rad, United States), and the quality was checked by agarose gel electrophoresis (Invitrogen, United States) with the GelRed^™^ nucleic acid gel stain (Goldbio, United States). We pooled three DNA samples from different replicates of the same treatments after DNA extraction for DGGE. Nested PCR was used to increase the resolution yield of DGGE. The highly variable V3 region of bacterial 16S rRNA gene was amplified as described by Xu et al. (2012) with a GC clamp (40 bp adhere to the 5' end) incorporated at the 5' end in primer 341F. The ITS1 region of the fungal 18S rRNA gene was amplified as described by Yu et al. (2013) with GC clamp (40 bp adhere to the 5' end) in primer ITS1-f. The second round of PCR yielded amplicons of bacteria and fungi that were approximately 190 bp and 250 bp, respectively. The primers used in this study can be found in Supplementary Table 1.

#### Denaturing Gradient Gel Electrophoresis and Sequence Analyses

PCR products were analyzed by 1.0% (w/v) agarose gel electrophoresis, stained with GelRed^™^, and visualized under UV light. The obtained PCR products were stored at −20°C for DGGE.

Nest PCR products were used for the DGGE analysis as described by Xu et al. (2012). The DCode^™^ Universal Mutation Detection System (Bio-Rad, CA, United States) was used for DGGE analysis. 40 μl of bacterial and fungal nested-PCR products per sample was loaded into an 8% (w/v) polyacrylamide (37.5:1 acrylamide/bio-acrylamide) gel containing a linear denaturing gradient of 35 to 80% for bacteria and 40 to 65% for fungi, where 100% denaturing acrylamide was defined as containing 7 M urea and 40% formamide (Muyzer et al., 1993). The gel was run for 10 min at 140 V, after which the voltage was lowered to 80 V for an additional 13 h in 1× TAE buffer at a constant temperature of 60°C. Gels were stained using the GelRed^™^ (Goldbio, United States), and gel images were digitally captured using the Gel Doc^™^XR System (Bio-Rad, United States). Rhizosphere microbial rRNA gene sequences of prominent DGGE bands were purified using the Universal DNA Purification Kit (Tiangen Biotech Co., Ltd., Beijing, China). Purified PCR products were cloned into the plasmid pGEM-T vector following the standard protocol of the pGEM-T Cloning Kit (Tiangen Biotech Co., Ltd., Beijing, China), and the ligation products were transformed into *Escherichia coli* (strain DH5a). Positive clones were identified based on blue-white screening. Cloned inserts were checked by PCR amplification using primers ITS1-F and ITS2 for fungi and 341f and 534r for bacteria (Xu et al., 2012; Yu et al., 2013). The rRNA genes obtained from the polyacrylamide gel were sequenced (Shanghai Sangon Biological Engineering Technology & Services Co., Ltd., China).

To confirm that the rhizospheric microbial rRNA gene sequences were of bacterial and fungal origin, obtained sequences were analyzed by basic local alignment search tool (BLAST) and compared with sequences deposited in the GenBank database at the NCBI. Phylogenetic relationships of the rhizosphere microbiota were analyzed by constructing phylogenetic trees which contained the sequences obtained by us and database reference sequences. All of these sequences were edited and trimmed manually using the BioEdit software (version 7.0.9.0). The neighbor-joining trees were constructed by using MEGA version 7.05. To determine the support for each clade and assess the reliability of the branching pattern, bootstrap analysis was performed using 1,000 replications.

### Data Analysis

The pattern and intensity of bands were analyzed by the Quantity One software 4.62 (Bio-Rad, United States). The presence and absence of the bands in DGGE profiles were coded as binary data. Principal component analysis (PCA) was chosen to infer the multivariate relationship between treatment factors and rhizosphere microbial communities. PCA was performed using Canoco (version 4.5, Centre for Biometry, Wageningen, Netherlands), and the Monte Carlo permutation test with 499 replicates was permuted using cyclic shifts. According to the number and intensity of bands in DGGE profiles, species richness (*S*), Shannon-Weiner index (*H*'), and Evenness index (*E*) were calculated as described by Yu et al. (2013):

$$H^{\prime }=-\sum_{i=1}^{s}Pi\ln Pi=-\sum_{i=1}^{s}Pi\ln\left(Ni/N)\ln(Ni/N\right)$$

$$E_{h}=\frac{H}{H_{\max}}=\frac{H}{\ln S}$$

where *Ni* is the peak density of the ith band, *N* is the total peak density of all bands in a lane, and *S* is the total band number in a lane (Xu et al., 2012; Ban et al., 2017).

Microbial biomass carbon, soil enzyme activities, cumulative disease incidence, and mortality were analyzed using SPSS (Version 16.0, International Business Machines Corp., Chicago, IL, United States). Cumulative disease incidence and mortality were analyzed using chi-square tests. C mic and soil enzyme activity were subjected to two-way and one-way analysis of variance after checking that normally distributed, respectively (Duncan’s test, *p* < 0.05). A Mantel test was conducted to compare the dissimilarity matrices of fungi and bacteria using XLStat 7.5 (Addinsoft, NY, United States), at probability level of 0.05 based on 10,000 permutations (Xu et al., 2012).

## Results

### Influence of Exogenous ECMF/DSE Inoculation on Disease Severity Caused by PWN Infection

**Figure 1:**
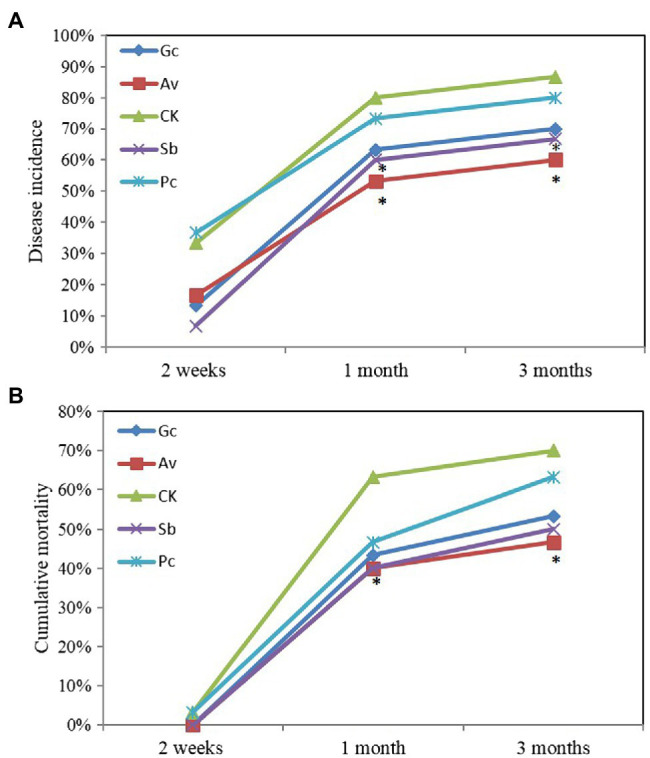
Cumulative disease incidence **(A)** and mortality **(B)** of ECMF and DSE *Pinus tabulaeformis* seedlings after inoculated with PWN. Av and Sb represent *P. tabulaeformis* seedlings were inoculated with ECMF of *Amanita vaginata* and *Suillus bovinus*, respectively; Gc and Pc represent the *P. tabulaeformis* seedlings were inoculated with DSE of *Gaeumannomyces cylindrosporus* and *Paraphoma chrysanthemicola*, respectively; and CK as the control group. The asterisk represents *p* value < 0.05 using chi-square test.

Wilting and yellowing of needles, both symptoms of PWD, appeared after 2 weeks of PWN inoculation. More than 40% of pine seedlings died between 2 weeks and 1 month after PWN inoculation. In contrast, after 3 months, the disease incidence and mortality of the control group were 86.7 and 70.0%, respectively (Figure 1). The disease incidence of Av and Sb treatments were significantly lower than the control group at 1 and 3 months, respectively (Figure 1A). Av treatment significantly reduced the mortality of *P. tabulaeformis* compared to the control group (Figure 1B). However, other exogenous ECMF/DSE treatments slightly reduced the disease severity of *P. tabulaeformis* compared with the control group (Figure 1).

### Effects of Exogenous ECMF/DSE Inoculation on Rhizosphere Microbial Biomass and Soil Enzymatic Activities Suffered From PWN Infection

**Figure 2:**
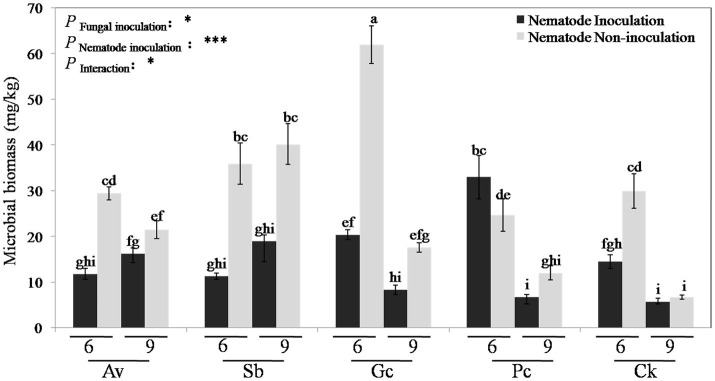
Rhizosphere soil microbial biomass carbon of different treatments. Av, Sb, Gc, and Pc mean pine seedlings inoculated with *A. vaginata*, *Suillus bovinus*, *G. cylindrosporus*, and *P. chrysanthemicola*, CK as control group. Six and nine mean seedling 6 and 9 months after nematode inoculation. The data are means ± SD (*n* = 3). Different lowercase letters indicate significant differences at *p* < 0.05, ^*^p < 0.05; and ^***^p < 0.001.

The C mic in the rhizosphere of *P. tabulaeformis* was measured at 6 and 9 months after PWN inoculation. Two-way ANOVA showed that the C mic content was significantly (*p* < 0.05) affected by the fungal inoculation, PWN inoculation, and the interaction between them (Figure 2). Except for the Pc treatment, the biomass carbon of *P. tabulaeformis* inoculated with PWN was significantly lower than that of the non-inoculated group. With PWN infection, the C mic of Av, Sb, Gc, and control treatments decreased by 45.03, 60.16, 63.81, and 44.76%, respectively, while Pc treatment increased by 8.17%. The microbial carbon contents in the rhizosphere of Av, Sb, Gc, and Pc treatments were 38.83, 107.51, 116.76, and 0.12% higher than that in the control group, respectively. With PWN inoculation, the microbial carbon contents of Av, Sb, Gc, and Pc treatments were 38.16, 49.67, 42.11, and 96.05% higher than that of the control group, respectively (Figure 2). These data showed that PWN infection decreased the rhizosphere C mic of *P. tabulaeformis* while exogenous ECMF/DSE inoculation promoted the accumulation of C mic. Rhizosphere soil enzyme activities of *P. tabulaeformis* were also measured and analyzed 6 and 9 months after PWN inoculation. The soil enzymatic activities of invertase, urease, and dehydrogenase were in some cases reduced due to the infection of PWN, in some cases (Supplementary Table 2). However, exogenous ECMF/DSE inoculation had no obvious effect on the soil enzymatic activities of *P. tabulaeformis* (Supplementary Table 2).

### Effects of Exogenous ECMF/DSE Inoculation on Rhizosphere Fungal and Bacterial Diversity Index Suffered From PWN Infection

**Figure 3:**
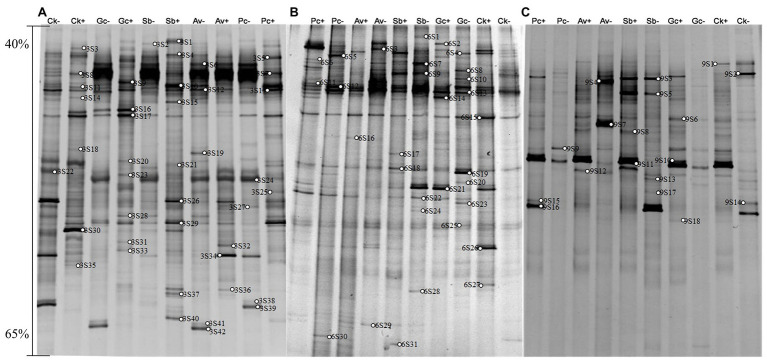
DGGE pattern of nested PCR-amplified rRNA gene fragments of fungi from *P. tabulaeformis* rhizosphere soils (**A**-**C** represent rhizosphere soils were sampled at 3, 6, and 9 months after PWN inoculation) of different ECMF and DSE inoculation treatments after PWN inoculation. Lanes labeled Gc, Sb, Av, and Pc in **A**-**C** represent *P. tabulaeformis* seedlings were inoculated with *G. cylindrosporus*, *Suillus bovinus*, *A. vaginata*, and *P. chrysanthemicola*, respectively; Ck as control group. “+” and “−” represent *P. tabulaeformis* seedlings were inoculated with PWN and were not inoculated with PWN, respectively. The linear gradient used was from 40 to 65% denaturant. The bands labeled 3S1-3S42 in **A**, 6S1-6S31 in **B**, and 9S1-9S18 in **C** were selected and excised from the DGGE gels and used for cloning and sequencing.

**Figure 4:**
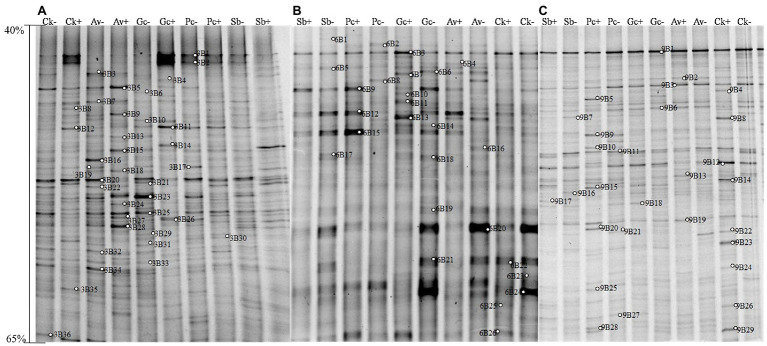
DGGE pattern of nested PCR-amplified rRNA gene fragments of bacteria from *P. tabulaeformis* rhizosphere soils (**A**-**C** represent rhizosphere soils were sampled at 3, 6, and 9 months after PWN inoculation) of different ECMF and DSE inoculation treatments after PWN inoculation. Lanes labeled Gc, Sb, Av, and Pc in **A**-**C** represent *P. tabulaeformis* seedlings were inoculated with *G. cylindrosporus*, *S. bovines*, *A. vaginata*, and *P. chrysanthemicola*, respectively; Ck as control group. “+” and “−” represent *P. tabulaeformis* seedlings were inoculated with PWN and were not inoculated with PWN, respectively. The linear gradient used was from 40 to 65% denaturant. The bands labeled 3B1-3B36 in **A**, 6B1-6S26 in **B**, and 9B1-9B29 in **C** were selected and excised from the DGGE gels and used for cloning and sequencing.

**Figure 5:**
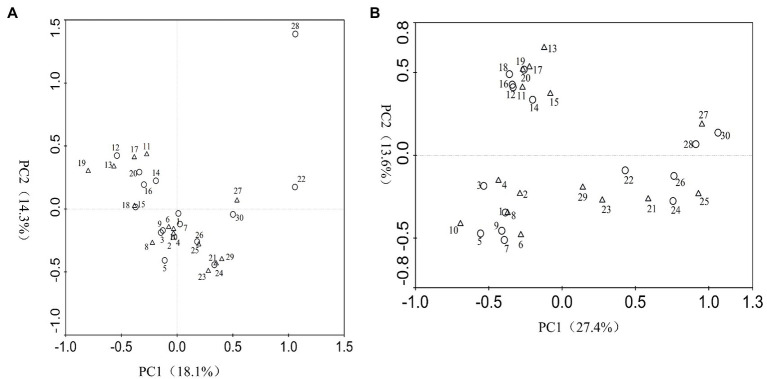
Principal components analysis (PCA) of fungi **(A)** and bacteria **(B)** species generated from DGGE band patterns. Circle represents pine seedlings non-inoculated with PWN, and triangle represents pine seedlings inoculated with PWN. 1~10, 11~20, and 21~30 represent samples from *P. tabulaeformis* seedlings inoculation of *A. vaginata*, *S. bovines, G. cylindrosporus*, *P. chrysanthemicola*, and control treatments at 3, 6, and 9 months after PWN infection, respectively. PC1, principal component 1; PC2, principal component 2.

According to the DGGE separation principle, each band in the DGGE map represents a single fungal group or operational taxonomic unit. The number of electrophoretic bands on each lane represents the species richness of the sample, and the intensity of each band represents the relative abundance of the species. The DGGE map analysis showed that the fungal community composition differed both among the different inoculation treatments and different sampling time. Overall, the DGGE map analysis showed that species richness (*S'*) and diversity (*H'*) of most treatments declined as culture duration increased both with and without PWN infection (Figures 3, 4 and Table 1). At 6 and 9 months after PWN inoculation, the diversity (*H'*) and richness(*S*) indices of most rhizosphere fungal communities of *P. tabulaeformis* had declined. Unexpectedly, and with the exception of the Av treatment, the diversity (*H'*) and richness (*S*) indices of rhizosphere fungi communities had increased in each treatment at 3 months after PWN inoculation (Table 1). As further confirmation of these changes through time, principal components analysis (PCA) showed that rhizosphere soil samples were clustered by sampling date (3, 6, and 9 months) especially for bacterial communities (Figure 5). At 9 months after PWN infection, fungal species richness (*S'*) and diversity (*H'*) in exogenous ECMF/DSE (Av, Sb, and Gc) inoculations were higher than that of control group (Table 1). Furthermore, PCA could not distinguish soil samples of rhizosphere fungal communities treated with and without PWN infection, which may be the result of artificial inoculation of exogenous ECMF/DSE (Figure 5A). However, inoculation of exogenous ECMF/DSE had at most, only a slight effect on bacterial community structure (Table 1 and Figure 4). In conclusion, these data indicate that after 9 months, inoculation of exogenous fungi (ECMF/DSE) reduced the influence of PWN and helped maintain the stability of fungal communities in the rhizosphere of *P. tabulaeformis*.

**Table 1:** Shannon-Wiener index (*H'*), richness (*S*), and evenness (*Eh*) of *Pinus tabulaeformis* rhizosphere bacteria and fungi in the different treatments at 3, 6, and 9 months after PWN inoculation.

	Index	months	Ck−	Ck+	Av−	Av+	Sb−	Sb+	Pc−	Pc+	Gc−	Gc+
Fungi	*S*	3	39	41	33	31	24	41	27	34	27	36
		6	24	28	22	22	28	25	29	26	31	24
		9	23	13	23	15	24	18	16	12	21	26
	*H'*	3	3.277	3.385	3.070	3.068	2.807	3.381	2.834	3.180	2.876	3.251
		6	2.789	3.039	2.818	2.81	2.912	2.968	3.052	2.892	3.065	2.827
		9	2.733	2.211	2.913	2.202	2.877	2.566	2.310	2.091	2.683	2.647
	*E_h_*	3	0.894	0.912	0.878	0.893	0.883	0.91	0.86	0.902	0.873	0.907
		6	0.878	0.912	0.912	0.91	0.874	0.922	0.907	0.888	0.893	0.889
		9	0.872	0.862	0.929	0.813	0.905	0.888	0.84	0.843	0.881	0.813
Bacteria	*S*	3	35	33	35	36	37	25	31	28	37	41
		6	24	28	29	26	22	26	28	26	29	28
		9	20	32	22	23	33	29	27	29	28	23
	*H'*	3	3.532	3.446	3.522	3.544	3.579	3.178	3.391	3.223	3.557	3.683
		6	3.111	3.305	3.332	3.234	3.037	3.225	3.322	3.207	3.334	3.302
		9	2.809	3.301	2.925	2.981	3.37	3.214	3.144	3.236	3.181	2.984
	*E_h_*	3	0.994	0.986	0.991	0.989	0.991	0.987	0.988	0.967	0.985	0.992
		6	0.979	0.992	0.989	0.993	0.983	0.99	0.997	0.984	0.990	0.991
		9	0.938	0.952	0.946	0.951	0.964	0.955	0.954	0.961	0.955	0.952

*Av, Sb Gc, and Pc represent P. tabulaeformis seedlings were inoculated with Amanita vaginata, Suillus bovinus, Gaeumannomyces cylindrosporus, and Paraphoma chrysanthemicola, respectively; Ck as control group. + and* − *represent P. tabulaeformis seedlings were inoculated with PWN and were not inoculated with PWN, respectively. Three, six, and nine represent P. tabulaeformis seedlings were inoculated with PWN for3, 6, and 9 months, respectively. S represents species richness, H' represents Shannon-Weiner index, and E_h_ represents evenness index*.

### Phylogenetic Analyses and Microbial Taxon Identification

**Figure 6:**
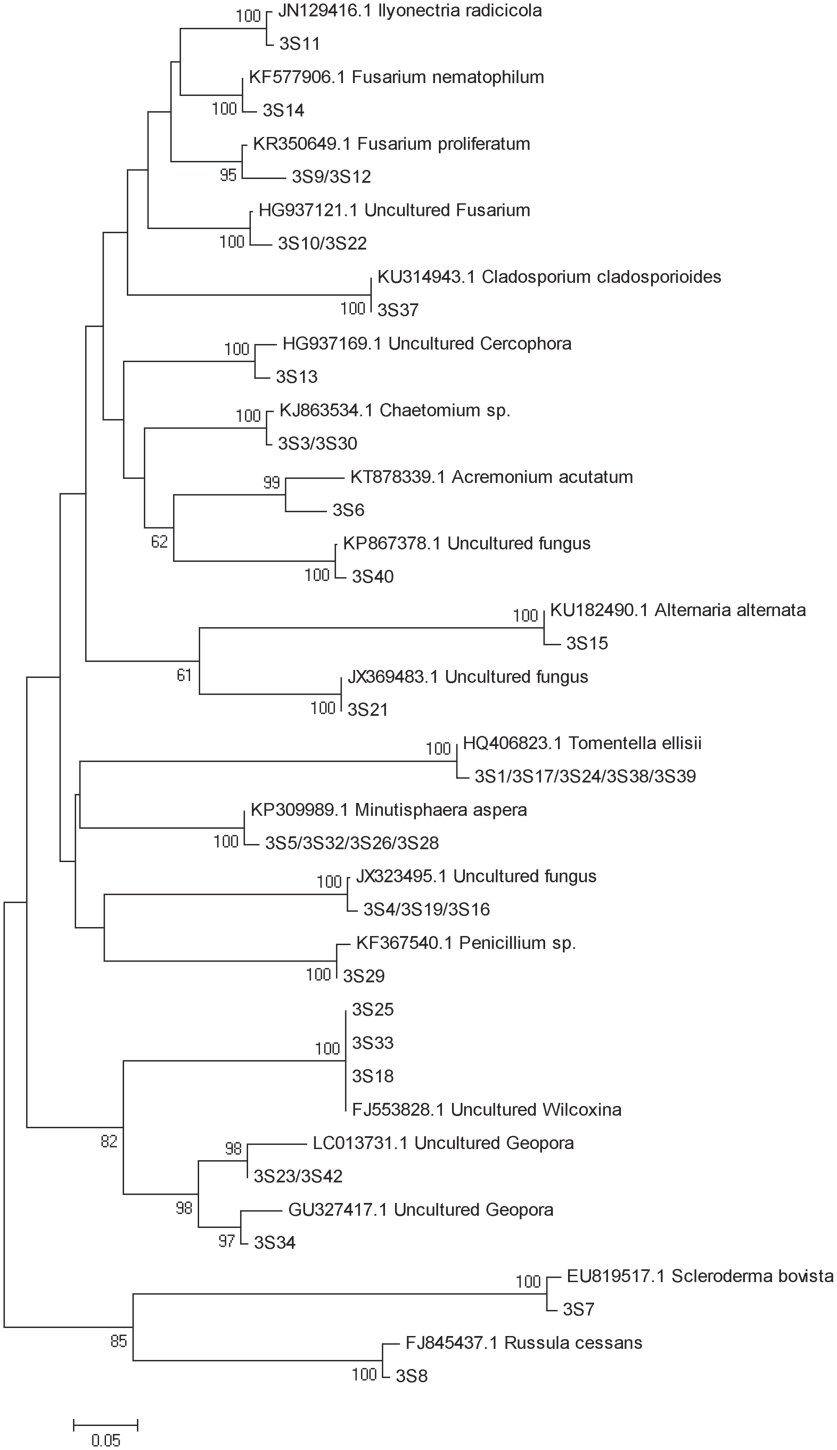
Neighbor-joining phylogenetic tree of fungi at 3 months after PWN infection. According to the distribution of ITS rRNA gene fragments in DGGE gel electrophoresis in Figure 3A, some common and unique bands’ sequences were recovered from the DGGE gels and performed phylogenetic analysis with reference sequences. Bootstrap values (out of 1,000) were shown when they exceed 60% (Same as following).

**Figure 7:**
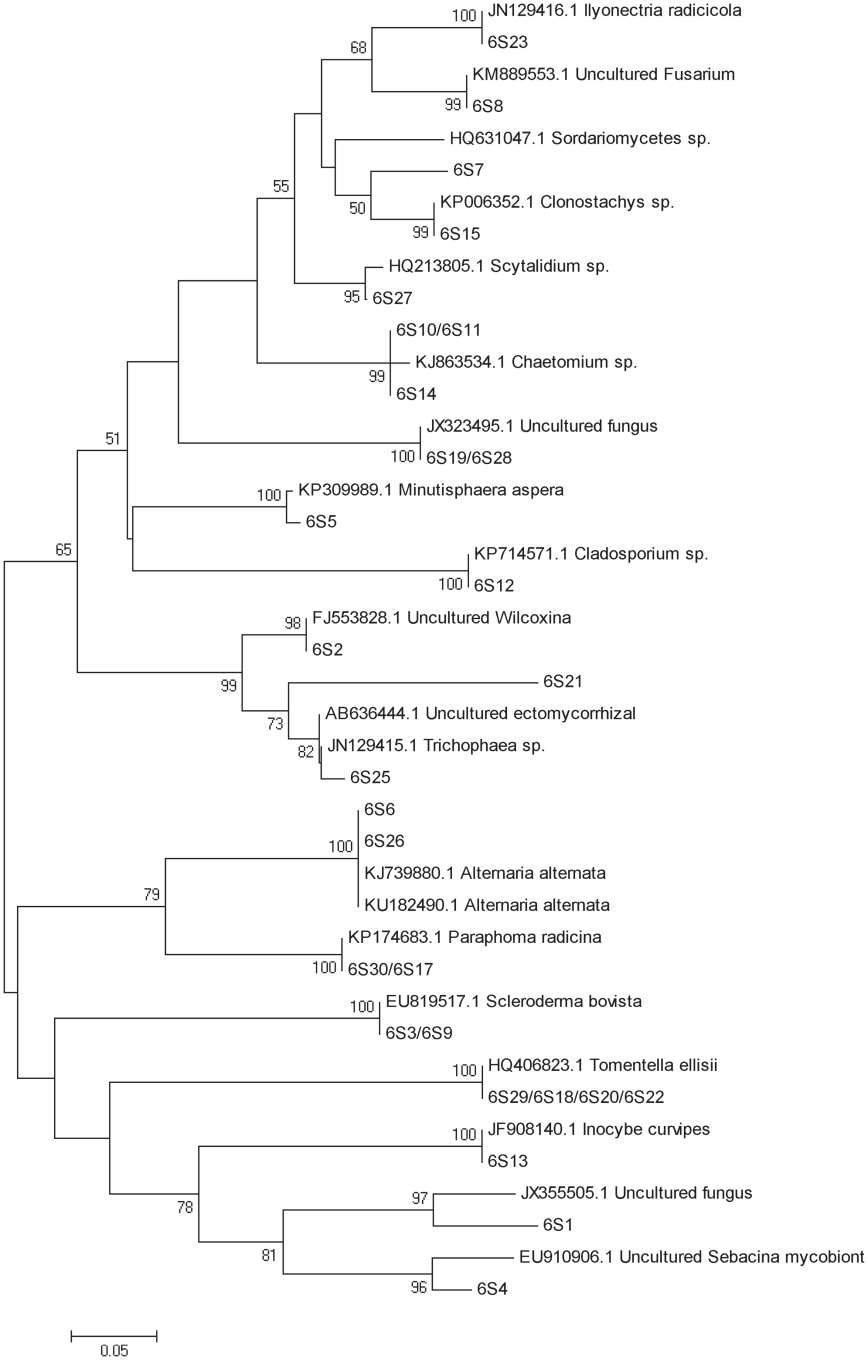
Neighbor-joining phylogenetic tree of fungi at 6 months after PWN infection.

**Figure 8:**
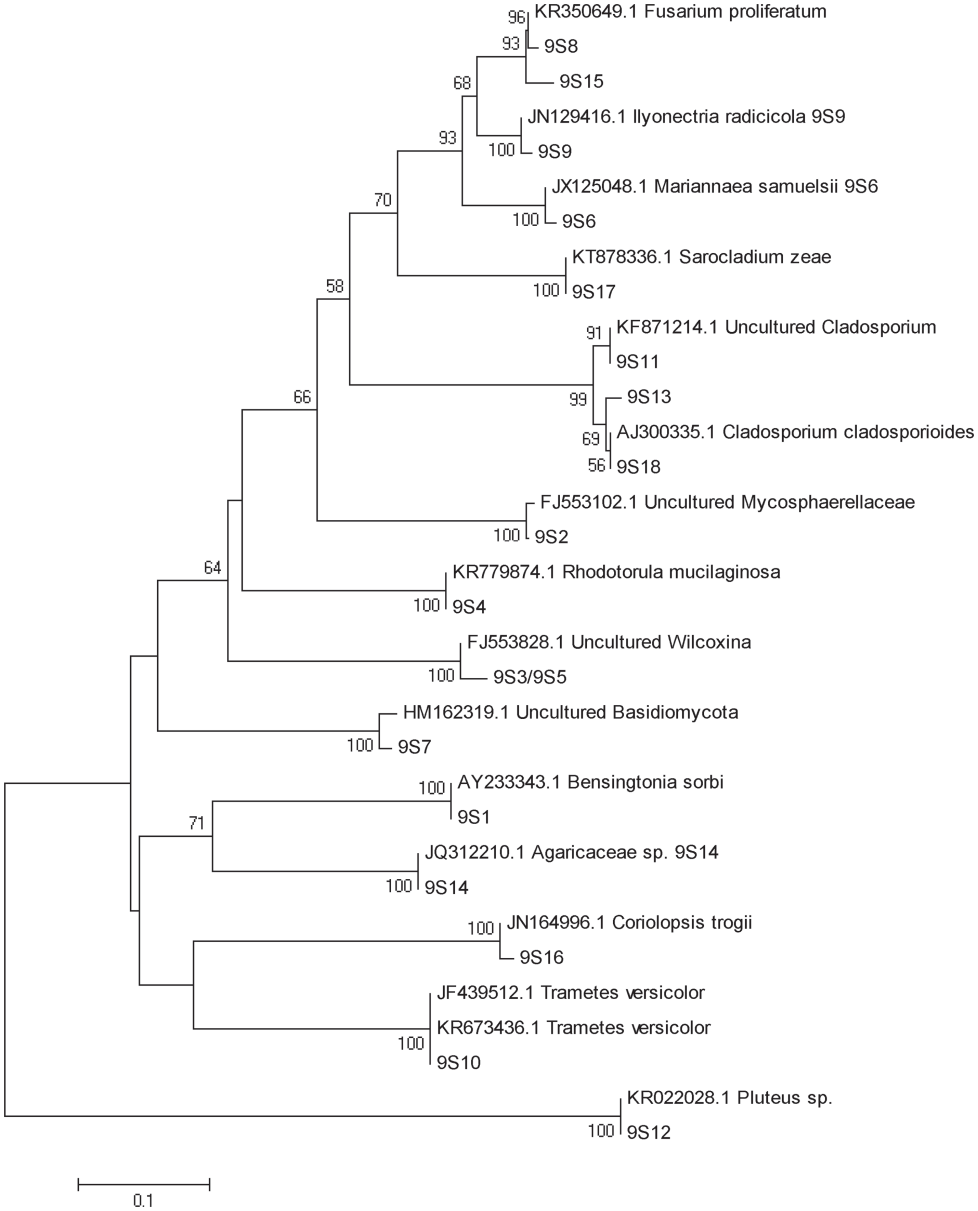
Neighbor-joining phylogenetic tree of fungi at 9 months after PWN infection.

A total of 77 bands of fungi were excised, cloned, and sequenced successfully, of which 35, 24, and 18 were successfully sequenced from rhizosphere soil of 3, 6, and 9 months after PWN inoculation, respectively (Figure 3; Supplementary Table 3). The BLAST showed that all the sequences were highly similar to sequences from members of Ascomycota and Basidiomycota (Supplementary Table 3). Based on the closest BLAST matches and phylogenetic analysis, 20, 19, and 15 different taxa were identified in rhizosphere soil of 3, 6, and 9 months after PWN inoculation, respectively (Figures 6–8). 22 of 77 bands belonged to ECMF, with 15 of those belonged to endophytes, 2 being saprophytes, 2 being pathogens, 1 being a helper, and the remaining being of unknown classification (Figure 3; Supplementary Table 3). All inoculated ECMF/DSE can recruit beneficial microbes, especially for beneficial fungi (Table 2; Supplementary Tables 3 and 4). Sb, Av, Gc, and Pc recruited 6, 4, 7, and 6 groups of beneficial fungi, respectively, such as *Tomentella ellisii*, *Wilcoxina mikolae*, and uncultured *Wilcoxina*. *T. ellisii* was the most frequently recruited fungal group in all exogenous ECMF/DSE treatments (Table 2).

**Table 2:** The recruited beneficial microbes by exogenous ectomycorrhizal fungi/dark septate endophyte (ECMF/DSE).

	Beneficial Fungi							Beneficial Bacteria	
Sb	3S1	3S24	3S33	3S38	3S39	9S5			3B23
Av	3S15	3S24	6S2	9S4				3B16	3B23
Gc	3S1	3S7	3S15	3S24	3S33	6S2	6S20		3B23
Pc	3S1	3S15	3S24	3S38	3S39	6S20			3B23

*Av, Sb, Gc, and Pc represent P. tabulaeformis seedlings were inoculated with A. vaginata, S. bovines, G. cylindrosporus, and P. chrysanthemicola, respectively. Numbers of 3, 6, and 9 before capital letter “S” or “B” represent beneficial fungus or beneficial bacteria of P. tabulaeformis rhizosphere soils sampled at 3, 6, and 9 months after PWN inoculation excised from the DGGE gels. Numbers after capital letter “S” or “B” represent bands number mark in the DGGE gels*.

Likewise, 35, 26, and 29 bacterial bands were sequenced from rhizosphere soil of 3, 6, and 9 months after PWN inoculation, respectively (Figure 4). Based on the closest BLAST matches and phylogenetic analysis, the main groups of rhizosphere bacteria are Proteobacteria, Actinobacteria, and Acidobacteria (Supplementary Table 4). DGGE mapping and phylogenetic analysis revealed that the inoculation with exogenous fungi enriched and recruited the beneficial bacteria in the rhizosphere of *P. tabulaeformis*, such as *Thauera* sp. and *Mesorhizobium* sp. (Supplementary Table 4 and Supplementary Figures 1–3). However, fewer recruited beneficial bacteria were recruited than beneficial fungi (Supplementary Table 3).

## Discussion

Many studies have shown that the colonization of ECMF and DSE could confer pathogen resistance to host plants (Mandyam and Jumpponen, 2005; Zhang and Tang, 2012; Zhang et al., 2017; Chu et al., 2019; Poveda et al., 2020), and this effect was observed in the present study, as well. Exogenous inoculation with ECMF/DSE delayed the development of PWD symptoms and reduced disease severity of *P. tabulaeformis* (Figure 1). After 1 month of PWN infection, the cumulative disease incidence of Av, Sb, Gc, and Pc treatments was 26.7, 20, 16.7, and 6.7% lower than the control group, respectively. And the cumulative mortality of Av, Sb, Gc, and Pc treatment was 26.6, 23.3, 20, and 10% lower than the control group (Figure 1).

Soil enzyme activities and microbial biomass can be indicators of soil health (Alkorta et al., 2003; Dotaniya et al., 2019). And “good soil” can improve plant viability and disease resistance too (Mendes et al., 2011; Berendsen et al., 2012; Carrión et al., 2018; Kumar and Verma, 2019). We studied the effect of ECMF and DSE inoculation on rhizosphere microbial community structure, C mic and soil enzyme activities of PWN infected *P. tabulaeformis* seedlings. Although ECMF inoculation can improve rhizosphere soil enzymatic activities (Lu et al., 2016), in our study, exogenous ECMF/DSE inoculation seemed to have no obvious effect on soil enzymatic activities. One explanation is that soil enzymatic activities might not be as sensitive in the relatively stable greenhouse conditions and small isolated pots as they would be in natural conditions. Consistent with prior results (Shi et al., 2015), PWN infection significantly decreased C mic of the rhizosphere. However, exogenous ECMF/DSE inoculation increased (*p* < 0.01) the rhizosphere microbial biomass (Figure 2). Zhang et al. (2010) also found that ECMF inoculation increased the amount of C mic in the rhizosphere of *P. tabulaeformis*, from 49.6 μgg^−1^ in the control group to 134.02 μgg^−1^ (Zhang et al., 2010). In this study, the microbial carbon contents of Av, Sb, Gc, and Pc were 38.16, 49.67, 42.11, and 96.05% higher than PWN infection treatments, respectively (Figure 2). These data indicate that exogenous ECMF/DSE inoculation alleviated the reduction of C mic caused by PWD. Thus, the microenvironment of *P. tabulaeformis* might be improved by exogenous ECMF/DSE inoculation under PWD interference.

Under natural conditions, PWD interference can alter the composition and diversity of fungal communities (Akema and Futai, 2005; Ugawa et al., 2009; Chu et al., 2016, 2018; Nakashima et al., 2016) and bacteria (Mabuhay and Nakagoshi, 2012; Shi et al., 2015) in pine forests. Using DGGE, we analyzed the effects of PWN infection on the diversity and community structure of rhizosphere fungi and bacteria in the greenhouse. Because DGGE is not sensitive and visualizes only the dominant taxa (Muyzer et al., 1993; Xu et al., 2012; Nimnoi et al., 2017), so the fungal and bacterial communities mentioned in this paper actually referred to the dominant fungal and bacterial communities, and the same situation was applied for the microbial diversity analysis. We were able to determine how fungal and bacterial taxa and communities varied under different treatments based on diversity indices. It is not surprising that the rhizosphere microbial diversity and richness of *P. tabulaeformis* decreased gradually in the small isolated pots in which *P. tabulaeformis* was growing. Interestingly, inoculation with exogenous fungi (ECMF/DSE) reduced fungal species diversity at an early stage, which may be due to increased competition between the original fungi and the exogenous fungi. In addition, consistent with the results of Akema and Futai (2005), PWN inoculation reduced diversity (*H'*) and richness (*S'*) of the rhizosphere fungal community. Interestingly, we found that rhizosphere fungal diversity was higher at an early stage of PWN infection. It may be due to the lack of nutrient supply in the roots due to the early infection of PWN, which reduced the competitiveness of root fungi and increased the complexity of rhizosphere microbe (Mendes et al., 2011; Philippot et al., 2013; Olanrewaju et al., 2019). Furthermore, compared with the control group, the reduction of rhizosphere fungal species richness (*S'*) and diversity (*H'*) was alleviated by exogenous ECMF/DSE (Av, Sb, and Gc) inoculation after 9 months (Table 1). PCA could not distinguish soil samples of rhizosphere fungal communities treated with and without PWN infection probably due to the beneficial effect of exogenous ECMF/DSE inoculation. Thus, exogenous ECMF/DSE inoculation treatments promoted rhizosphere functional resilience, which may be one of the reasons that inoculation of ECMF/DSE improved the resistance of *P. tabulaeformis* to PWD.

In contrast to fungi, bacterial diversity was only slightly impacted by infection of PWN. However, the DGGE fingerprints and PCA distinguished bacterial communities of rhizosphere soil collected at 3, 6, and 9 months after PWN infection, implying that the culture duration was a major factor affecting bacterial community structure. Nimnoi et al. (2017) also found that the inoculants (*Streptomyces galilaeus*) did not influence bacterial community structure, whereas stages of plant growth mainly caused the shift in bacterial community structure. However, Shi et al. (2015) found that bacterial diversity of *P. massoniana* forests was reduced by PWD. DGGE applied in these previous studies and ours were short of biological replication. Additional replication is needed to draw stronger conclusions.

Mycorrhizae not only help the host plant to improve nutrient conditions, improve disease resistance and stress resistance (Smith and Read, 2010), but also recruit and enrich other microorganisms that are beneficial to themselves and/or plants (Cameron et al., 2013; Gupta and Aggarwal, 2018). Our study showed that with or without PWN infection, inoculation with exogenous ECMF/DSE increased the relative abundance rhizosphere beneficial fungi and bacteria (Figures 3, 4; Supplementary Tables 2 and 3), such as *Thauera* sp. *Rhizobium* and increases the relative abundance of ECMF in the rhizosphere of *P. tabulaeformis* (Chen et al., 2006; Barriuso et al., 2008; Buscardo et al., 2010; Geml et al., 2010; Huang et al., 2012; Wang et al., 2018). These beneficial fungi and bacteria are widely reported to plant growth promotion (PGP) traits through various mechanisms including biologic nitrogen fixation, solubilization of minerals, chelation of iron, and secretion of plant growth hormones (Smith and Read, 2010; Volpiano et al., 2019; Romero-Perdomo et al., 2021). In addition, they showed various biocontrol traits, such as plays a role in plant disease suppression, induces host systemic resistance (Pieterse et al., 2014; Meena et al., 2017; Singh et al., 2017; Tonelli et al., 2020). These results also contribute to improving the resistance of *P. tabulaeformis* to PWD. In addition, *T. ellisii* was the dominant species among recruited fungi in this study, possibly because species in the genus *Tomentella* (Thelephoraceae) belong to the most frequent and widespread ECMF in temperate and boreal forests (Jakucs et al., 2005).

In summary, our study suggests that exogenous inoculation of ECMF/DSE can maintain the stability of rhizosphere fungal and bacterial communities, slow down the reduction of microbial biomass, and alleviate the influences of PWD on rhizosphere fungal and bacterial communities. In addition, ECMF/DSE can increase the relative abundance of beneficial rhizosphere fungi and bacteria. These effects might explain how ECMF and DSE improve *P. tabulaeformis’* resistance to PWD.

## Data Availability Statement

The original contributions presented in the study are included in the article/Supplementary Material, and further inquiries can be directed to the corresponding author.

## Author Contributions

CW and MT designed and supervised this study. HC, HW, and ZL performed the experiments. HC and YZ analyzed the data and drafted the manuscript. MT, ZL, DD, and HC revised the manuscript. All authors read and approved manuscript for submission.

## Conflict of Interest

The authors declare that the research was conducted in the absence of any commercial or financial relationships that could be construed as a potential conflict of interest.

## Publisher’s Note

All claims expressed in this article are solely those of the authors and do not necessarily represent those of their affiliated organizations, or those of the publisher, the editors and the reviewers. Any product that may be evaluated in this article, or claim that may be made by its manufacturer, is not guaranteed or endorsed by the publisher.
