# Supplementary material for: Inoculation With Ectomycorrhizal Fungi and Dark Septate Endophytes Contributes to the Resistance of Pinus spp. to Pine Wilt Disease
Source: Front Microbiol. 2021 Aug 6;12:687304. doi: 10.3389/fmicb.2021.687304 (PMC8377431; doi:10.3389/fmicb.2021.687304)
Supplement: Supplementary file 1 [file Data_Sheet_1.docx]

Supplementary Material

**Table 1:** Primers used for nested PCR amplifcation of soil fungal and bacterial communities in the rhizosphere of *P. tabulaeformis*

| **Taxa** | **Nested PCR** | **Primer name** | **Primer sequence (5’-3’)** |
| --- | --- | --- | --- |
| **Fungi** | First round | ITS1-f | CTTGGTCATTTAGAGGAAGTAA |
|  |  | ITS4 | TCCTCCGCTTATTGATATGC |
|  | Second round | ITS1-f-Clamp | Clamp-CTTGGTCATTTAGAGGAAGTAA |
|  |  | ITS2 | GCTGCGTTCTTCATCGATGC |
| **Bacteria** | First round | fD1 | AGAGTTTGATCCTGGCTCAG |
|  |  | rD1 | ACGGTTACCTTGTTACGACTT |
|  | Second round | 341f-Clamp | Clamp-CCTACGGGAGGCAGCAG |
|  |  | 534r | ATTACCGCGGCTGCTGG |

Note:GC clamp sequence: 5’-CGCCCGCCGCGCGCGGCGGGCGGGGCGGGGGCACGGGGGG-3’.

Table 2 Effects of pine wood nematode infection on soil enzymatic activites of different fungi inoculation treatments

| Treatments | | Invertase  （mg Glc g^-1^h^-1^） | Urease  (mg NH_3_-N g^-1^h^-1^) | Phosphatase  (mg PhOH g^-1^h^-1^) | Dehydrogenase（mg TPF g^-1^h^-1^） |
| --- | --- | --- | --- | --- | --- |
| 6 | Av+ | 0.08±0.01g | 0.70±0.1abcdef | 0.0012±0.00017abcd | 0.83±0.04cdefg |
|  | Av- | 0.20±0.03abc | 0.52±0.25abcdef | 0.0012±0.00014abcd | 0.91±0.1cdef |
| 9 | Av+ | 0.08±0.00g | 0.35±0.03ef | 0.0010±0.00015bcd | 0.44±0.02g |
|  | Av- | 0.21±0.04ab | 0.87±0.08abcd | 0.0013±0.00002ab | 0.97±0.14bcde |
| 6 | Sb+ | 0.22±0.06a | 0.28±0.08f | 0.0011±0.00012abcd | 0.82±0.04cdefg |
|  | Sb- | 0.20±0.03abc | 0.44±0.04cdef | 0.0012±0.00015abc | 1.18±0.07abc |
| 9 | Sb+ | 0.12±0.01defg | 0.71±0.11abcdef | 0.0013±0.00002ab | 0.63±0.05efg |
|  | Sb- | 0.16±0.01bcd | 0.85±0.15abcd | 0.0013±0.00011a | 0.77±0.1defg |
| 6 | Ck+ | 0.17±0.03abcd | 0.80±0.21abcde | 0.0009±0.00005d | 0.99±0.12bcde |
|  | Ck- | 0.15±0.01cde | 0.25±0.02f | 0.0010±0.00004cd | 0.87±0.07cdef |
| 9 | Ck+ | 0.07±0.01g | 0.30±0.02f | 0.0011±0.00022abcd | 0.47±0.04g |
|  | Ck- | 0.16±0.03bcd | 0.97±0.02a | 0.0010±0.00014bcd | 0.84±0.07cdefg |
| 6 | Gc+ | 0.20±0.04abc | 0.45±0.08bcdef | 0.0010±0.00008bcd | 0.62±0.09efg |
|  | Gc- | 0.16±0.05bcd | 0.55±0.15abcdef | 0.0012±0.00023abcd | 1.51±0.16a |
| 9 | Gc+ | 0.09±0.00fg | 0.93±0.25ab | 0.0012±0.0001abcd | 0.70±0.14defg |
|  | Gc- | 0.16±0.02bcd | 0.82±0.01abcde | 0.0010±0.00008cd | 0.84±0.07cdefg |
| 6 | Pc+ | 0.15±0.04bcde | 0.41±0.1def | 0.0009±0.00009d | 1.07±0.19bcd |
|  | Pc- | 0.16±0.06bcd | 0.90±0.4abc | 0.0012±0.00024abcd | 1.34±0.33ab |
| 9 | Pc+ | 0.12±0.01defg | 0.66±0.03abcdef | 0.0011±0.00016abcd | 0.57±0.03fg |
|  | Pc- | 0.11±0.03efg | 0.83±0.01abcde | 0.0010±0.00029cd | 0.66±0.02efg |

Note: Av, Sb Gc and Pc represent *P. tabulaefomis* seedlings were inoculated with *Amanita vaginata*, *Suillus bovines,Gaeumannomyces cylindrosporus* and *Paraphoma chrysanthemicola* respectively; Ck as control group. + and - represent *P. tabulaefomis* seedlings were inoculated with PWN and were not inoculated with PWN, respectively. 6 and 9 represent *P. tabulaefomis* seedlings were inoculated with PWN for 6 and 9 months respectively. Numbers stained in red represent significant reduction of soil enzymatic activites. Significant differences between means were determined by Duncan's test (P＜0.05). Data expressed as mean ± SD (n=3).

Table 3 Rhizosphere fungal sequences of the DGGE bands, their nearest BLAST matches of 3, 6, 9 months after PWN infection

| DGGE Band | Phyla | Accession no. | Nearest BLAST matches | Category | Identity (%) | References |
| --- | --- | --- | --- | --- | --- | --- |
| 3S3 | Ascomycota | MZ412626 | KJ863534*Chaetomium* sp. | UN | 98 | - |
| 3S30 | Ascomycota | MZ412627 | KJ863534*Chaetomium* sp. | UN | 98 | - |
| 3S5 | Ascomycota | MZ412628 | KP309989*Minutisphaera aspera* | - | 93 | - |
| 3S28 | Ascomycota | MZ412629 | KP309989*Minutisphaera aspera* | - | 97 | - |
| 3S32 | Ascomycota | MZ412630 | KP309989*Minutisphaera aspera* | - | 99 | - |
| 3S26 | Ascomycota | MZ412631 | KP235789*Uncultured Humicola* | - | 99 | - |
| 3S6 | Ascomycota | MZ412632 | KT878339*Acremonium acutatum* | Pathogen | 96 | Williams et al.(1987) |
| 3S9 | Ascomycota | MZ412633 | KR350649*Fusarium proliferatum* | Endophyte | 100 | Cheng et al. (2008) |
| 3S12 | Ascomycota | MZ412634 | KU534855*Uncultured Fusarium* | - | 100 | - |
| 3S10 | Ascomycota | MZ412635 | KM889552*Uncultured Fusarium* | - | 99 | - |
| 3S22 | Ascomycota | MZ412636 | HG937121Uncultured *Fusarium* | UN | 97 | - |
| 3S11 | Ascomycota | MZ412637 | JN129416*Ilyonectria radicicola* | Endophyte | 99 | Kwaśna et al. (2016) |
| 3S13 | Ascomycota | MZ412638 | HG937169Uncultured Cercophora | UN | 98 | - |
| 3S14 | Ascomycota | MZ412639 | KF577906*Fusarium nematophilum* | Endophyte | 99 | Su et al. (2014) |
| 3S15 | Ascomycota | MZ412640 | KU182490*Alternaria alternata* | Endophyte | 100 | Guo et al. (2004) |
| 3S25 | Ascomycota | MZ412641 | FJ553828Uncultured *Wilcoxina* | ECMF | 98 | Egger et al. (1990) |
| 3S18 | Ascomycota | MZ412642 | KT447181*Wilcoxina mikolae* | ECMF | 99 | Egger et al. (1990) |
| 3S33 | Ascomycota | MZ412643 | KT447181*Wilcoxina mikolae* | ECMF | 100 | Egger et al. (1990) |
| 3S23 | Ascomycota | MZ412644 | FM206427Geopora sp. | UN | 90 | - |
| 3S42 | Ascomycota | MZ412645 | FM206427Geopora sp. | UN | 89 |  |
| 3S29 | Ascomycota | MZ412646 | KF367540*Penicillium* sp. | UN | 99 | - |
| 3S34 | Ascomycota | MZ412647 | GU327417Uncultured *Geopora* | UN | 97 | - |
| 3S37 | Ascomycota | MZ412648 | KU314943*Cladosporium cladosporioides* | DSE | 99 | Ban et al. 2012 |
| 3S1 | Basidiomycota | MZ412649 | HQ406823*Tomentella ellisii* | ECMF | 99 | Buscardo et al. (2010) |
| 3S17 | Basidiomycota | MZ412650 | HQ406823*Tomentella ellisii* | ECMF | 98 | Buscardo et al. (2010) |
| 3S24 | Basidiomycota | MZ412651 | HQ406823*Tomentella ellisii* | ECMF | 99 | Buscardo et al. (2010) |
| 3S38 | Basidiomycota | MZ412652 | HQ406823*Tomentella ellisii* | ECMF | 100 | - |
| 3S39 | Basidiomycota | MZ412653 | HQ406823*Tomentella ellisii* | ECMF | 97 | - |
| 3S7 | Basidiomycota | MZ412654 | EU819517*Scleroderma bovista* | ECMF | 98 | Chen et al. (2006) |
| 3S8 | Basidiomycota | MZ412655 | FJ845437*Russula cessans* | ECMF | 98 | Geml et al. (2010) |
| 3S4 | - | MZ412656 | JX323495Uncultured fungus | UN | 99 | - |
| 3S16 | - | MZ412657 | JX323495Uncultured fungus | UN | 100 |  |
| 3S19 | - | MZ412658 | JX323495Uncultured fungus | UN | 99 |  |
| 3S21 | - | MZ412659 | JX369483Uncultured fungus | UN | 100 | - |
| 3S40 | - | MZ412660 | KP867378Uncultured fungus | UN | 97 | - |
| DGGE Band | Phyla | Accession no. | Nearest BLAST matches | Category | Identity (%) | References |
| 6S2 | Ascomycota | MZ412667 | FJ553828Uncultured *Wilcoxina* | ECMF | 97 | Egger et al. (1990) |
| 6S5 | Ascomycota | MZ412670 | KP309989*Minutisphaera aspera* | - | 100 | - |
| 6S6 | Ascomycota | MZ412671 | KJ739880*Alternaria alternata* | Endophyte | 100 | Guo et al. (2004) |
| 6S10 | Ascomycota | MZ412662 | KJ863534*Chaetomium* sp. | UN | 98 | - |
| 6S12 | Ascomycota | MZ412677 | KP714571*Cladosporium* sp. | - | 100 | - |
| 6S15 | Ascomycota | MZ412679 | KP006352*Clonostachys* sp. | UN | 99 | - |
| 6S23 | Ascomycota | MZ412661 | JN129416*Ilyonectria radicicola* | Endophyte | 100 | Kwaśna et al. (2016) |
| 6S25 | Ascomycota | MZ412690 | JN129415*Trichophaea* sp. | UN | 90 | - |
| 6S26 | Ascomycota | MZ412672 | KU182490*Alternaria alternata* | Endophyte | 100 | Guo et al. (2004) |
| 6S27 | Ascomycota | MZ412674 | HQ213805*Scytalidium* sp. | UN | 96 | - |
| 6S3 | Basidiomycota | MZ412668 | EU819517*Scleroderma bovista* | ECMF | 99 | Chen et al. (2006) |
| 6S9 | Basidiomycota | MZ412666 | EU819517*Scleroderma bovista* | ECMF | 100 | Chen et al. (2006) |
| 6S4 | Basidiomycota | MZ412669 | EU910906Uncultured *Sebacina mycobiont* | ECMF | 96 | Urban et al. (2003) |
| 6S13 | Basidiomycota | MZ412678 | JF908140*Inocybe curvipes* | ECMF | 99 | Huang et al. (2012) |
| 6S20 | Basidiomycota | MZ412687 | HQ406823*Tomentella ellisii* | ECMF | 97 | Buscardo et al. (2010) |
| 6S18 | Basidiomycota | MZ412684 | HQ406823*Tomentella ellisii* | ECMF | 99 | Buscardo et al. (2010) |
| 6S22 | Basidiomycota | MZ412688 | HQ406823*Tomentella ellisii* | ECMF | 100 | Buscardo et al. (2010) |
| 6S29 | Basidiomycota | MZ412680 | HQ406823*Tomentella ellisii* | ECMF | 98 | Buscardo et al. (2010) |
| 6S21 | - | MZ412682 | AB636444Uncultured ectomycorrhizal | ECMF | 97 | - |
| 6S1 | - | MZ412665 | JX355505Uncultured fungus | UN | 97 | - |
| 6S7 | Ascomycota | MZ412673 | HQ631047*Sordariomycetes* sp. | UN | 96 | - |
| 6S8 | Ascomycota | MZ412676 | KM889553Uncultured *Fusarium* | UN | 99 | - |
| 6S28 | - | MZ412675 | JX323495Uncultured fungus | UN | 100 |  |
| 6S19 | - | MZ412685 | JX323495Uncultured fungus | UN | 99 | - |
| DGGE Band | Phyla | Accession no. | Nearest BLAST matches | Category | Identity (%) | References |
| 9S2 | Ascomycota | MZ412691 | FJ553102Uncultured Mycosphaerellaceae | UN | 98 | - |
| 9S6 | Ascomycota | MZ412692 | JX125048*Mariannaea samuelsii* | UN | 99 | - |
| 9S8 | Ascomycota | MZ412693 | KR350649*Fusarium proliferatum* | Endophyte | 100 | Cheng et al. (2008) |
| 9S15 | Ascomycota | MZ412694 | KR350649*Fusarium proliferatum* | Endophyte | 99 | Cheng et al. (2008) |
| 9S9 | Ascomycota | MZ412695 | JN129416*Ilyonectria radicicola* | Endophyte | 99 | Kwaśna et al. (2016) |
| 9S11 | Ascomycota | MZ412696 | KF871214Uncultured *Cladosporium* | UN | 100 | - |
| 9S13 | Ascomycota | MZ412697 | AJ300335*Cladosporium cladosporioides* | DSE | 99 | Ban et al. 2012 |
| 9S18 | Ascomycota | MZ412699 | AJ300335*Cladosporium cladosporioides* | DSE | 100 | Ban et al. 2012 |
| 9S17 | Ascomycota | MZ412698 | KT878336*Sarocladium zeae* | Endophyte | 99 | Falade et al. (2016) |
| 9S1 | Basidiomycota | MZ412700 | AY233343*Bensingtonia sorbi* | UN | 99 | - |
| 9S3 | Basidiomycota | MZ412701 | FJ553828Uncultured *Wilcoxina* | ECMF | 99 | Egger et al. (1990) |
| 9S5 | Basidiomycota | MZ412703 | KT447181*Wilcoxina mikolae* | ECMF | 99 | Egger et al. (1990) |
| 9S4 | Basidiomycota | MZ412702 | KR779874*Rhodotorula mucilaginosa* | Helper | 99 | Buzzini et al. (2005) |
| 9S7 | Basidiomycota | MZ412704 | HM162319Uncultured Basidiomycota | UN | 97 | - |
| 9S10 | Basidiomycota | MZ412705 | JF439512*Trametes versicolor* | Pathogen | 100 | Dittmann et al. (2002) |
| 9S12 | Basidiomycota | MZ412706 | KR022028*Pluteus* sp. | Saprophyte | 98 | Menolli et al. (2010) |
| 9S14 | Basidiomycota | MZ412707 | JQ312210*Agaricaceae* sp. | UN | 99 | - |
| 9S16 | Basidiomycota | MZ412708 | JN164996*Coriolopsis trogii* | Saprophyte | 99 | Wojewoda and Karasiński  (2010) |

Note:The bands' sequences labeled 3S1-3S42, 6S1-6S31 and 9S1-9S18 were selected and excised from the DGGE gelselectrophoresis in Figure3."ECMF" represent the sequence of the band was identified as ectomycorrhizal fungi which are shown in red, "DSE" represent the sequence of the band was identified as dark separate endophytic fungi which are show in blue,"Helper"represent the sequence of the band was identified asmycorrhiza helper microorganisms, "Endophyte" represent the sequence of the band was identified as endophytic fungi which are shown in green, "Pathogen" represent the sequence of the band was identified as pathogenic fungi, "Saprophyte" represent the sequence of the band was identified assaprophytic fungi, "UN" represent the sequence of the band was not be identified, "-" represent the sequence of the band was not found the reference.

Table 4 Rhizosphere bacterial sequences of the DGGE bands, their nearest BLAST matches of 3, 6, 9 months after PWN infection

| DGGE Band | Phylum | Accession no. | Identity (%) | Nearest BLAST matches |
| --- | --- | --- | --- | --- |
| 3B3 | Actinobacteria | MZ388998 | 99 | KU560315Yonghaparkia sp. |
| 3B5 | Acidobacteria | MZ388975 | 100 | DQ828868Uncultured Acidobacteria bacterium |
| 3B7 | Acidobacteria | MZ388977 | 99 | HM438210Uncultured Acidobacteriaceae bacterium |
| 3B10 | Actinobacteria | MZ388980 | 100 | AJ555205uncultured Actinobacteridae bacterium |
| 3B14 | Actinobacteria | MZ388984 | 99 | JQ793536Uncultured actinobacterium |
| 3B31 | Acidobacteria | MZ388999 | 99 | AY150892Uncultured Acidobacteriales bacterium |
| 3B4 | Bacteroidetes | MZ388974 | 98 | HF564253Uncultured Bacteroidetes bacterium |
| 3B6 | Chloroflexi | MZ388976 | 99 | AM936176Uncultured Anaerolineae bacterium |
| 3B9 | Chloroflexi | MZ388979 | 99 | EU297607Uncultured Chloroflexi bacterium |
| 3B12 | Chloroflexi | MZ388982 | 100 | JQ861377Uncultured Chloroflexi bacterium |
| 3B22 | Chlorobi | MZ388991 | 97 | AM935163Uncultured Chlorobi bacterium |
| 3B32 | Chloroflexi | MZ389000 | 99 | EF188666Uncultured Chloroflexi bacterium |
| 3B1 | Firmicutes | MZ388971 | 99 | KX890233*Enterococcus casseliflavus* |
| 3B30 | Gemmatimonadetes | MZ388998 | 98 | KF182857Uncultured Gemmatimonadetes bacterium |
| 3B8 | Proteobacteria | MZ388978 | 100 | KM219107*Pseudomonas* sp. |
| 3B13 | Proteobacteria | MZ388983 | 99 | JQ433745Uncultured proteobacterium |
| 3B16 | Proteobacteria | MZ388986 | 100 | KX770732*Mesorhizobium* sp. |
| 3B17 | Proteobacteria | MZ388981 | 94 | EU300412Uncultured Xanthomonadaceae bacterium |
| 3B18 | Proteobacteria | MZ388987 | 100 | LC016985Uncultured alpha proteobacterium |
| 3B19 | Proteobacteria | MZ388988 | 100 | KU341396*Sphingomonas yunnanensis* |
| 3B21 | Proteobacteria | MZ388990 | 100 | KU177235*Pseudomonas* sp. |
| 3B23 | Proteobacteria | MZ409029 | 99 | LN555098Uncultured Burkholderiales |
| 3B24 | Planctomycetes | MZ388992 | 95 | JN825600Uncultured Planctomycetales bacterium |
| 3B25 | Proteobacteria | MZ388993 | 99 | FN679067Uncultured *Dechloromonas* sp. |
| 3B26 | Proteobacteria | MZ388994 | 98 | KT322546Uncultured delta proteobacterium |
| 3B27 | Proteobacteria | MZ388995 | 100 | KU752847*Pseudomonas putida* |
| 3B28 | Proteobacteria | MZ388996 | 99 | EU299260Uncultured beta proteobacterium |
| 3B33 | Proteobacteria | MZ389001 | 99 | KX083679*Agrobacterium* sp. |
| 3B35 | Proteobacteria | MZ389003 | 98 | KX083679*Agrobacterium* sp. |
| 3B34 | Proteobacteria | MZ389002 | 99 | EF651098Uncultured *Rubrivivax* sp. |
| 3B36 | Proteobacteria | MZ409030 | 99 | JQ861847Uncultured *Brucella* sp. |
| 3B2 | - | MZ388972 | 99 | JX080258Uncultured bacterium |
| 3B11 | - | MZ388981 | 99 | KJ613005Uncultured bacterium |
| 3B15 | - | MZ388985 | 97 | KP154486Uncultured bacterium |
| 3B20 | - | MZ388989 | 100 | JQ640325Uncultured bacterium |
| DGGE Band | Phylum | Accession no. | Identity (%) | Nearest BLAST matches |
| 6B2 | Actinobacteria | MZ389006 | 99 | KX881425*Paenarthrobacter nicotinovorans* |
| 6B12 | Actinobacteria | MZ389016 | 100 | KP823695*Dactylosporangium luteum* |
| 6B17 | Actinobacteria | MZ389019 | 100 | AY150951Uncultured actinobacterium |
| 6B24 | Actinobacteria | MZ389026 | 100 | KU052207Uncultured actinobacterium |
| 6B26 | Acidobacteria | MZ389029 | 97 | AM934954Uncultured Acidobacteria bacterium |
| 6B3 | Bacteroidetes | MZ389007 | 98 | JQ723651Uncultured Sphingobacteriales bacterium |
| 6B7 | Bacteroidetes | MZ389010 | 99 | KU305719*Chitinophaga pinensis* |
| 6B14 | Bacteroidetes | MZ389004 | 100 | KT369895*Pedobacter caeni* |
| 6B25 | Bacteroidetes | MZ389028 | 98 | JX500612Uncultured Algoriphagus sp. |
| 6B20 | Chloroflexi | MZ389020 | 98 | JX473225Uncultured Anaerolineae bacterium |
| 6B1 | Firmicutes | MZ389005 | 99 | LC140805Uncultured *Lactococcus* sp. |
| 6B11 | Firmicutes | MZ389015 | 99 | KX890471*Bacillus megaterium* |
| 6B5 | Proteobacteria | MZ389012 | 100 | KT452782*Rhizobacter* sp. |
| 6B6 | Proteobacteria | MZ389009 | 99 | KX881459*Pseudomonas* sp. |
| 6B15 | Proteobacteria | MZ389018 | 99 | KX881459*Pseudomonas* sp. |
| 6B8 | Proteobacteria | MZ389011 | 97 | KM100469Uncultured Desulfuromonadales bacterium |
| 6B9 | Proteobacteria | MZ389013 | 99 | LN680484uncultured Burkholderiales bacterium |
| 6B10 | Proteobacteria | MZ389014 | 99 | LC025325*Duganella* sp. |
| 6B13 | Proteobacteria | MZ389017 | 98 | KM624175Uncultured *Legionella* sp. |
| 6B16 | Proteobacteria | MZ389022 | 99 | LC094483Rhodobacteraceae bacterium |
| 6B18 | Proteobacteria | MZ389020 | 99 | KX454117*Sphingopyxis alaskensis* |
| 6B19 | Proteobacteria | MZ389021 | 100 | KF145700Uncultured *Pseudomonas* sp. |
| 6B22 | Proteobacteria | MZ389027 | 96 | KU563136*Thauera* sp. |
| 6B23 | Proteobacteria | MZ389023 | 100 | JQ401916Uncultured alpha proteobacterium |
| 6B4 | Verrucomicrobia | MZ389008 | 99 | LN833280*Luteolibacter* sp. |
| 6B21 | Verrucomicrobia | MZ389021 | 99 | KX010383Uncultured *Opitutus* sp. |
| DGGE Band | Phylum | Accession no. | Identity (%) | Nearest BLAST matches |
| 9B3 | Acidobacteria | MZ389032 | 98 | KM656203Uncultured Acidobacteria bacterium |
| 9B11 | Acidobacteria | MZ389033 | 98 | KM656203Uncultured Acidobacteria bacterium |
| 9B4 | Actinobacteria | MZ389035 | 99 | KF287259Uncultured Actinobacterium |
| 9B26 | Acidobacteria | MZ409031 | 99 | EF457496Uncultured Acidobacteria bacterium |
| 9B28 | Actinobacteria | MZ389039 | 98 | JQ400598Uncultured Actinobacterium |
| 9B21 | Bacteroidetes | MZ389053 | 99 | JQ400917Uncultured Sphingobacteriales bacterium |
| 9B13 | Chloroflexi | MZ389047 | 99 | AY922002Uncultured Chloroflexi bacterium |
| 9B19 | Chloroflexi | MZ389052 | 99 | KC535150Uncultured Chloroflexi bacterium |
| 9B5 | Firmicutes | MZ389037 | 98 | EF664605Uncultured Firmicutes bacterium |
| 9B17 | Firmicutes | MZ389038 | 98 | EF664605Uncultured Firmicutes bacterium |
| 9B20 | Firmicutes | MZ389057 | 97 | LC140805Uncultured *Lactococcus* sp. |
| 9B1 | Gemmatimonadetes | MZ389030 | 99 | JN409176Uncultured Gemmatimonadetes bacterium |
| 9B2 | Gemmatimonadetes | MZ389031 | 99 | JQ071716Uncultured Gemmatimonadetes bacterium |
| 9B8 | Gemmatimonadetes | MZ389042 | 99 | HG325754Uncultured Gemmatimonadetes bacterium |
| 9B18 | Gemmatimonadetes | MZ389051 | 100 | EF612383Uncultured Gemmatimonadetes bacterium |
| 9B27 | Gemmatimonadetes | MZ389034 | 99 | KC747081Uncultured *Gemmatimonas* sp. |
| 9B6 | Proteobacteria | MZ389040 | 100 | EF072952Uncultured proteobacterium |
| 9B7 | Proteobacteria | MZ389041 | 100 | JX505035Uncultured Hyphomicrobiaceae bacterium |
| 9B9 | Proteobacteria | MZ389043 | 100 | JF904879*Pseudorhodoferax* sp. |
| 9B10 | Proteobacteria | MZ389044 | 97 | KR061379Uncultured *Hirschia* sp. |
| 9B12 | Proteobacteria | MZ389045 | 100 | KF145700Uncultured *Pseudomonas* sp. |
| 9B14 | Proteobacteria | MZ389048 | 100 | JF703331Uncultured beta proteobacterium |
| 9B16 | Proteobacteria | MZ389050 | 99 | JF703331Uncultured beta proteobacterium |
| 9B15 | Proteobacteria | MZ389049 | 100 | LN680484uncultured Burkholderiales bacterium |
| 9B22 | Proteobacteria | MZ389054 | 98 | AB696838*Azoarcus* sp. |
| 9B23 | Proteobacteria | MZ389055 | 98 | KT308446Uncultured *Thauera* sp. |
| 9B25 | Proteobacteria | MZ389056 | 99 | KX378896*Pseudomonas* sp. |
| 9B29 | Proteobacteria | MZ389046 | 100 | EF074179Uncultured *Rhodoplanes* sp. |
| 9B24 | Verrucomicrobia | MZ389036 | 97 | HM163278Verrucomicrobia bacterium |

Note: The bands' sequenceslabeled 3B1-3B36, 6B1-6S26 and 9B1-9B29 were selected and excised from the DGGE gelselectrophoresis in Figure4. "-" represent the sequence was not be identified.


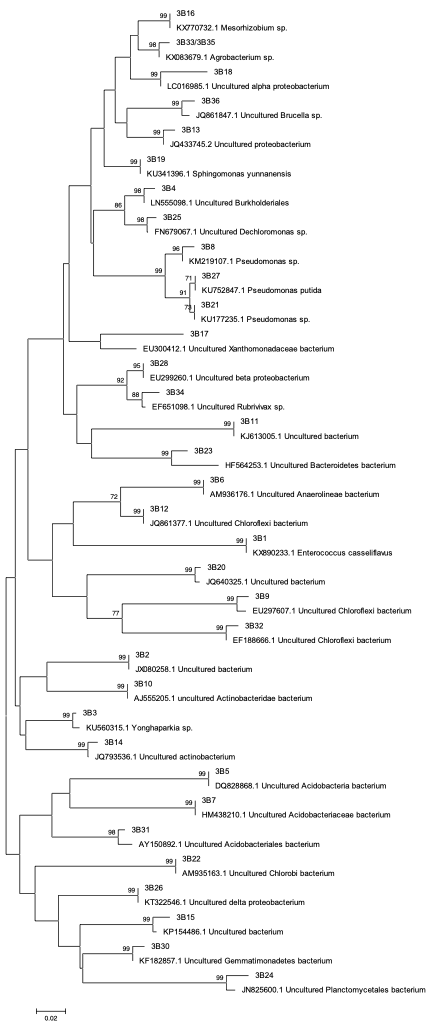


Figure1 Neighbor-joining phylogenetic tree of bacteria at 3 months after PWN infection. According to the distribution of rRNA gene fragments in DGGE gel electrophoresis in Figure4A,some common and unique bands' sequences were recovered from the DGGE gels and performed phylogenetic analysis with reference sequences. Bootstrap values (out of 1000)were shown when they exceed 60%.


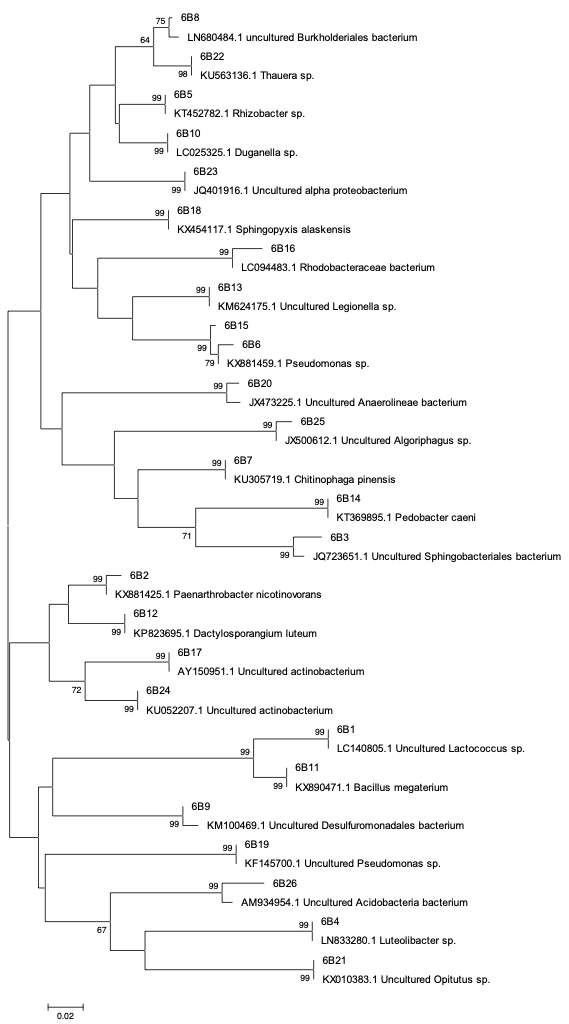


Figure2 Neighbor-joining phylogenetic tree of bacteria at 6 months after PWN infection. According to the distribution of rRNA gene fragments in DGGE gel electrophoresis in Figure4B,some common and unique bands' sequences were recovered from the DGGE gels and performed phylogenetic analysis with reference sequences. Bootstrap values (out of 1000)were shown when they exceed 60%.


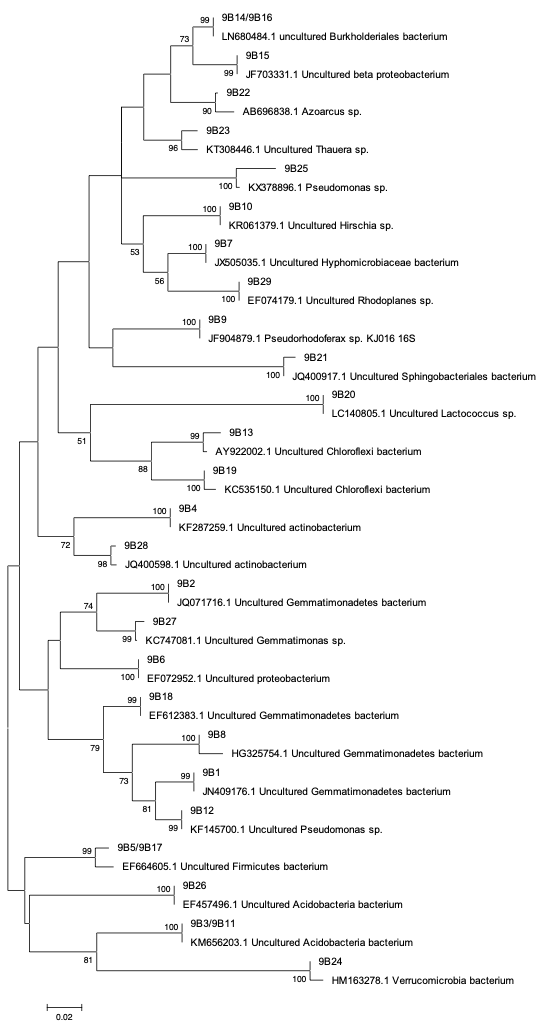


Figure3 Neighbor-joining phylogenetic tree of bacteria at 9 months after PWN infection. According to the distribution of rRNA gene fragments in DGGE gel electrophoresis in Figure4C,some common and unique bands' sequences were recovered from the DGGE gels and performed phylogenetic analysis with reference sequences. Bootstrap values (out of 1000)were shown when they exceed 60%

Data link:

<https://submit.ncbi.nlm.nih.gov/subs/?search=SUB9847805>and

<https://submit.ncbi.nlm.nih.gov/subs/?search=SUB9869536> for bacteria;

<https://submit.ncbi.nlm.nih.gov/subs/?search=SUB9844719>for fungus.

# References

Buscardo, E., Rodríguez-Echeverría, S., Martín, M. P., De Angelis, P., Pereira, J. S., and Freitas, H. (2010). Impact of wildfire return interval on the ectomycorrhizal resistant propagules communities of a Mediterranean open forest.*Fungal Biol.*114, 628-636. doi: 10.1016/j.funbio.2010.05.004

Buzzini, P., Gasparetti, C., Turchetti, B., Cramarossa, M. R., Vaughan-Martini, A., Martini, A., et al. (2005). Production of volatile organic compounds (VOCs) by yeasts isolated from the ascocarps of black (*Tuber melanosporum* Vitt.) and white (*Tuber magnatum* Pico) truffles.*Archives of microbiology*,84, 187-193. doi: 10.1007/s00203-005-0043-y

Chen, Y. L., Kang, L. H., Malajczuk, N., and Dell, B. (2006). Selecting ectomycorrhizal fungi for inoculating plantations in south China: effect of Scleroderma on colonization and growth of exotic *Eucalyptus globulus*, *E. urophylla*, *Pinus elliottii*, and *P. radiata*.*Mycorrhiza*16, 251-259. doi: 10.1007/s00572-006-0039-8

Cheng, Z. S., Tang, W. C., Su, Z. J., Cai, Y., Sun, S. F., Chen, Q. J., et al. (2008). Identification of mangrove endophytic fungus 1403 (*Fusarium proliferatum*) based on morphological and molecular evidence.*J. Forestry Res.* 19, 219. doi: 10.1007/s11676-008-0030-7

Dittmann, J., Heyser, W., and Bücking, H. (2002). Biodegradation of aromatic compounds by white rot and ectomycorrhizal fungal species and the accumulation of chlorinated benzoic acid in ectomycorrhizal pine seedlings.*Chemosphere*49, 297-306. doi: 10.1016/S0045-6535(02)00323-5

Egger, K. N., and Fortin, J. A. (1990). Identification of taxa of E-strain mycorrhizal fungi by restriction fragment analysis.*Canadian J. Botany*68, 1482-1488. doi: https://doi.org/10.1139/b97-869

Falade, T. D., Syed Mohdhamdan, S. H., Sultanbawa, Y., Fletcher, M. T., Harvey, J. J., Chaliha, M., and Fox, G. P. (2016). In vitro experimental environments lacking or containing soil disparately affect competition experiments of *Aspergillus flavus* and co-occurring fungi in maize grains.*Food Additives & Contaminants: Part A*,33, 1241-1253. doi: 10.1080/19440049.2016.1198048

Geml, J., Laursen, G. A., Herriott, I. C., McFarland, J. M., Booth, M. G., Lennon, N., et al. (2010). Phylogenetic and ecological analyses of soil and sporocarp DNA sequences reveal high diversity and strong habitat partitioning in the boreal ectomycorrhizal genus *Russula* (Russulales; Basidiomycota).*New Phytol*.187, 494-507. doi: 10.1111/j.1469-8137.2010.03283.x

Guo, L., Xu, L., Zheng, W. H., and Hyde, K. D. 2004. Genetic variation of *Alternaria alternata*, an endophytic fungus isolated from *Pinus tabulaeformis* as determined by random amplified microsatelites (RAMS). *Fungal Divers.* 16: 53-65.

Huang, J., Nara, K., Lian, C., Zong, K., Peng, K., Xue, S., and Shen, Z. (2012). Ectomycorrhizal fungal communities associated with Masson pine (*Pinus massoniana* Lamb.) in Pb-Zn mine sites of central south China. *Mycorrhiza* 22(8), 589-602. doi: 10.1007/s00572-012-0436-0

Kwaśna, H., Szewczyk, W., and Behnke-Borowczyk, J. (2016). Fungal root endophytes of *Q. uercus* robur subjected to flooding.*Forest Pathol.*46, 35-46. doi: 10.1111/efp.12212

Menolli Jr, N., Asai, T., and Capelari, M. (2010). Records and new species of Pluteus from Brazil based on morphological and molecular data.*Mycology*1, 130-153.doi: 10.1080/21501203.2010.493531

Su, H., Kang, J. C., Cao, J. J., Mo, L., and Hyde, K. D. (2014). Medicinal plant endophytes produce analogous bioactive compounds.*Chiang Mai J Sc*,41, 1-13.

Urban, A., Wei, M., and Bauer, R. (2003). Ectomycorrhizas involving sebacinoid mycobionts.*Mycol. Res.*107, 3-14.doi: 10.1017/S0953756202007116

Williams, M. A. J. (1987). Descriptions of pathogenic fungi and bacteria. Mycopathologia, 100, 169-190.

Wojewoda, W., Karasiński, D. (2010) Invasive macrofungi (Ascomycota and Basidiomycota) in Poland. In Biological Invasions in Poland; Mirek, Z., Ed.; W. Szafer Institute of Botany, Polish Academy of Sciences: Kraków, Poland, 2010; Volume 1, pp. 7–21
